# The Role of Psychiatry in the Management of Sexual Assault: A Case Series

**DOI:** 10.7759/cureus.13644

**Published:** 2021-03-01

**Authors:** Michelle Zaydlin, Linda Pérez-Laras, Linda Laras

**Affiliations:** 1 Psychiatry, University of Miami Miller School of Medicine/Jackson Memorial Hospital, Miami, USA; 2 Epidemiology and Public Health, Puerto Rico Health Justice Center/Centro Salud Justicia; San Juan Bautista School of Medicine, Caguas, PRI; 3 Obstetrics and Gynecology, Puerto Rico Health Justice Center/Centro Salud Justicia; Forensic Pediatric/Adolescent Gynecology, San Juan Bautista School of Medicine, Caguas, PRI

**Keywords:** child sexual abuse, multidisciplinary decision-making, mental health services, child abuse and neglect, access to healthcare, • access to healthcare and health outcomes of vulnerable populations, child and adolescent psychiatry, clinical forensic medicine, psychiatry and mental health

## Abstract

Victims of childhood sexual abuse are at an increased risk for a multitude of mental health conditions. While many children exhibit concerning behavioral changes following abuse, there is often a delay in identification and implementation of psychiatric services, resulting in worsening mental and physical health outcomes for victims. This case series aims to demonstrate the importance of multidisciplinary victim-centered and trauma-focused treatment including proactive psychiatric care. The review presents three cases of child victims of sexual abuse who received psychiatric care after their initial presentation to the Puerto Rico Health Justice Center (PRHJC). As evidenced by the following cases and extant literature, child victims of sexual abuse have an increased risk of severe mental health disorders. This indicates the importance of recognizing and understanding behavioral warning signs of childhood sexual abuse and the importance of psychiatric care as early as possible following disclosure.

## Introduction

Children who have experienced sexual violence are at an increased risk of developing mental health conditions when compared to children without a history of maltreatment [1,2]. In spite of the devastating short- and long-term psychological effects of violence on children, there remains limited access to interdisciplinary trauma-focused treatment centers that provide comprehensive forensic clinical care, including psychological, medical and educational services.

Individuals who experience trauma are at risk of developing symptoms of post-traumatic stress disorder (PTSD) including, but not limited to, nightmares, flashbacks, avoidance of memories, and increased arousal [3,4]. In addition to PTSD, other conditions strongly associated with childhood sexual abuse include: depression, anxiety, conversion disorder, borderline personality disorder, psychosis, eating disorders, self-injury, suicidal ideation and suicide attempts are also strongly associated with childhood sexual abuse [5-9]. Often, victims often meet the criteria for more than one of the aforementioned diagnoses [10]. This demonstrates that victims of childhood sexual abuse are at increased risk for multiple long-term mental illnesses requiring psychiatric care even long after the initial incident of abuse.

The objective of this case series is to emphasize the importance of incorporating early psychiatric intervention following a child’s disclosure of sexual violence. A delay in receiving adequate diagnosis and treatment can impact the child’s trauma and mood symptoms, ultimately leading to an increased need for subsequent psychiatric hospitalization and long-term treatment [5]. Early psychiatric intervention can dramatically impact the mental health of victims of child sexual abuse.

Here we present three cases of child victims of sexual violence. All three received psychiatric care after their initial presentation to the Puerto Rico Health Justice Center (PRHJC) located at the San Juan Bautista School of Medicine in Caguas, Puerto Rico. The PRHJC is a multidisciplinary victim-centered and trauma-focused program that offers clinical services for survivors of sexual violence.

Case information was obtained via a review of existing patient files from January 2018- January 2019. All participants were children who were victims of sexual violence in Puerto Rico who were treated at the PRHJC. Identifying information was removed for the protection of the patient’s privacy. The PRHJC and San Juan Bautista School of Medicine Review Board approved this study.

## Case presentation

Case 1

The first case considers an 11-year-old male who had been a victim of sexual assault including oral and penetrative anal intercourse prior to presentation at the PRHJC. While the male victim was unable to identify the date of the first incident of abuse, given the most recent dates of contact between the child and aggressor, it was stipulated that at least one year had passed between the last incident of abuse and the disclosure. It is believed that the time from the first incident of abuse to disclosure was a little over two years prior to presentation at the PRHJC. The victim initially disclosed the event to a family member and later to his mother. All the incidents of abuse occurred at aggressor's home.

Based on the mother’s report at the time of presentation to PRHJC, the child had begun to exhibit a variety of concerning behaviors over the two years. She reported that he showed aggressive behavior at school along with frequent tantrums at home and displayed difficulty following instructions, poor frustration tolerance, and irritability both at home and school. She also reported that he suffered from frequent nightmares, somniloquy, and bruxism.

Upon initial evaluation, it was reported that over the two years preceding presentation at the PRHJC, the victim had been suffering from the above-mentioned abrupt changes in mood and behavior. Following the assault, there was a history of one inpatient psychiatric hospitalization; however, the child was not diagnosed with a formal illness and not receive any long-term psychiatric care after discharge. He did follow up with psychotherapy; however, this was discontinued after a year for unclear reasons. 

Importantly, symptoms were identified both at home and school and despite being evaluated by physicians, nurses and therapists he deteriorated. At the time of disclosure and presentation to the PRHJC, two years following the assault, this victim was without appropriate psychiatric care or follow-up. This delay in identification and care likely contributed to the continued exacerbation of mood and behavioral symptoms. 

Case 2

This case considers a 13-year-old female victim of drug-facilitated sexual assault. At the time of presentation to the PRHJC, the victim was 17-years-old and four years had passed between the incident of abuse and the disclosure. The aggressor, in this case, was a family member and the incident occurred in the aggressor's home.

When the patient had access to the PRHJC, following the disclosure of the abuse, the adolescent reported significant emotional distress that began following the assault. She reported increased anxiety, insomnia, poor concentration, labile mood, and academic dysfunction. The victim's mother also confirmed these behavioral changes and reported that the adolescent also experienced frequent nighttime awakenings, nightmares, sleep talking, and bruxism and had begun to suffer from trichotillomania and nail-biting. School history, at the time of presentation to the PRHJC, was significant for a dramatic decrease in academic performance. 

As in the previous case, the child had been demonstrating significant symptoms across multiple settings. Similarly, several individuals were aware of the changes in the child’s conduct, yet opportunities for intervention were missed. Despite symptoms indicative of emotional distress, at the time of presentation to the PRHJC the child did not receive psychiatric care.

Case 3

Lastly, this case considers a 17-year-old female victim of childhood sexual abuse by her stepfather. The incident occurred two years before disclosure, and the abuse occurred within the victim’s home. 

Upon presentation to the PRHJC, this victim was exhibiting a multitude of psychiatrically concerning behaviors that began shortly following the abuse. Within the school setting the child had experienced significant academic decline, including failing one class, and in the year prior to presentation was no longer participating in previously enjoyed extra-curricular activities. At home, the child was noted to have poor sleep secondary to frequent nighttime awakening and excessive daytime fatigue. The victim’s mother reported that on multiple occasions, the child would become easily upset, leading her to lock herself in her room and isolate. During several of these instances, the victim had expressed wanting to take her own life. 

Again, the child experienced multiple worrisome behaviors leading to episodes of suicidal thoughts, yet no psychiatric services were received. It is notable that before presentation at the PRHJC the child had received a psychological evaluation due to her abrupt decline in academic performance; however, the warning signs of child sexual abuse continued unidentified, symptoms persisted, and appropriate care was not provided.

## Discussion

Through this case series, we emphasize the importance of providing early psychiatric care to children exposed to sexual violence, and to be on the lookout for possible indicators of child sexual abuse when a child manifests a change in behavior. In all three cases we observed that a delay in receiving adequate psychiatric diagnosis and treatment impacted the victim's mental health, leading to overall worse trauma and mood symptoms. 

Our findings are consistent with previous literature, that children who are victims of sexual abuse are at an increased risk for severe mental health outcomes, ranging from mood disorders to severe post-traumatic stress disorder [7-9,11]. As noted in the cases mentioned, many children who face traumatic experiences often go without psychiatric intervention following the event despite the onset of concerning behavioral symptoms. Recognizing child sexual abuse must include understanding concerning behaviors and emotional indicators. It is also important to note that the signs and symptoms of child sexual abuse are often present across settings. Thus, the child’s primary caregiver along with other individuals in the child’s life, including teachers and school counselors, should identify signs concerning abuse [12]. This team approach improves access to care and mental-health outcomes. 

## Conclusions

Utilizing a multidisciplinary victim-centered and trauma-focused approach that includes psychiatric services can decrease the negative impact of trauma on mental health. Therefore, we recommend including psychiatric care in the team approach of victim-focused care to minimize mental health crises experienced by trauma victims and improve collaboration amongst healthcare professionals, and, most importantly, improve outcomes for our patients.
